# The bud break process and its variation among local populations of boreal black spruce

**DOI:** 10.3389/fpls.2014.00574

**Published:** 2014-10-28

**Authors:** Sergio Rossi, Jean Bousquet

**Affiliations:** ^1^Département des Sciences Fondamentales, Université du Québec à ChicoutimiChicoutimi, QC, Canada; ^2^Chaire de Recherche du Canada en Génomique Forestière et Environnementale, Centre D'étude de la Forêt et Institut de Recherche en Biologie Intégrative et des Systèmes, Université LavalQuébec, QC, Canada

**Keywords:** adaptation, cold climate, genecology, genetic diversity, phenology, temperature

## Abstract

Phenology of local populations can exhibit adaptations to the current environmental conditions resulting from a close interaction between climate and genotype. The bud break process and its variations among populations were analyzed in greenhouse by monitoring the growth resumption in black spruce [*Picea mariana* (Mill.) BSP] seedlings originating from seeds of five stands across the closed boreal forest in Quebec, Canada. Bud break lasted 15 days and occurred earlier and quicker in northern provenances. Provenance explained between 10.2 and 32.3% of the variance in bud break, while the families accounted for a smaller but still significant part of the variance. The late occurrence of one phenological phase corresponded to a delayed occurrence of the others according to linear relationships. A causal model was proposed in the form of a chain of events with each phase of bud break being related to the previous and successive one, while no link was observed between non-adjacent phases. The adaptation of black spruce populations along the latitudinal gradient points toward a strategy based on rapid physiological processes triggered by temperature increase inducing high metabolic activity. The variation observed in bud break reflects an evolutionary trade-off between maximization of security and taking advantage of the short growing season. This work provides evidence of the phenological adaptations of black spruce to its local environmental conditions while retaining sizeable genetic diversity within populations. Because of the multigenic nature of phenology, this diversity should provide some raw material for adaptation to changing local environmental conditions.

## Introduction

In cold climates, most physiological processes of plants occur during a short lapse of time when the environmental factors, mainly temperature, are favorable to growth and reproduction. Thus, all meristems designated to flowering, fructification, and primary and secondary growth follow alternating periods of activity and rest, with annual cycles. The phenological phases bounding the growing season represent a trade-off between environmental constraints and resource availability, and identify the period of the year when resources can be acquired and used (Nord and Lynch, 2009). In ecosystems at high latitudes or altitude, the growing season is the result of an optimization between avoidance of frost damage and maximization of growth and carbon assimilation (Chuine, 2010).

The activity of meristems involves a number of biochemical processes resulting in a sequence of stages of development or maturation. These stages are identified according to morphological or anatomical changes and can last from a few days, in primary meristems, to several weeks, in the secondary meristem (Rossi et al., 2013; Rossi, 2014). A full understanding of the mechanisms of growth resumption in spring, a key adaptive trait in plants, is problematic because of the interaction of the abiotic (climate) and biotic (genotype) factors underlying this physiological process. Bud break is an example of phenological event of growth resumption that constitutes an intriguing and widely studied model trait composed by sequential events during which the embryonic shoots and leaves rapidly proliferate and emerge from the bud scales. Bud phenology integrates the effects of genotype and environmental conditions (Beaulieu et al., 2004; Rossi et al., 2009; Man and Lu, 2010). As natural selection tends to favor the genotypes that are better adapted to the local temperature conditions, some adaptive traits develop according to the gradual temperature change with latitude (Morgenstern, 1969). The timings of bud break are one of the traits that can change among populations along the latitude (Blum, 1988; Rossi, 2014). As a result, boreal tree species, particularly those with wide geographic distributions, show genetic variations at a regional scale in response to variation of climatic factors (Beaulieu et al., 2001; Andalo et al., 2005).

The phenology of buds and its variation over the last decades have assumed particular importance because shifts in phenological events have pointed out substantial changes in the environment (Anderson et al., 2013), or mismatches among species along food chains (Johnson et al., 2010). Synchronisms between timings of leafing and larval feeding of insect defoliators regulate outbreak occurrence and the survival strategy of hosts and parasites (Rossi et al., 2012b). In a context of possible assisted migration of species, detailed information on bud phenology and its structure may be crucial as seed transfer and artificial regeneration can benefit from ecotypes with specific adaptations to maintain plant biodiversity or forest productivity in a changing environment (Beaulieu and Bousquet, 2010; Prunier et al., 2013).

Another key issue regards the adaptation of local populations to the current conditions and their ability to adapt to future local conditions under climate change (Aitken et al., 2008). The present and next generations of local populations need the potential to adapt to the new or changing environmental conditions by means of phenotypic plasticity and genetic adaptation. While the former might be physiologically limited, the adaptation to wide modifications of the environment from one generation to the next will depend largely upon the amount of genetic variation within populations. Local populations are not genetically uniform but contain a certain amount of quantitative genetic variation relative to bud phenology (e.g., Li et al., 1993). In the context of climate change, most concern for forest tree species regards the genetic variation available for adaptive traits such as bud break and if the next generations will be able to cope with the new environmental conditions.

The aim of the present study was to investigate in detail the bud break process and to assess its variations among and within local populations. The following hypotheses were tested: (i) the developmental phases of the bud break process represent interconnected phenological events, (ii) the timings of bud break differ among provenances according to the latitude of the sites where seeds were collected, (iii) despite the differences among provenances, a sizeable variability in the timings of bud break may exist within populations. For attaining the objectives of the study, we monitored bud break in seedlings originating from seeds collected from five natural stands along an altitudinal/latitudinal range covering the entire closed black spruce [*Picea mariana* (Mill.) BSP] forest in Quebec, Canada. The seedlings of the five provenances were maintained under the same environmental conditions, which allowed variations among local populations and their families to be quantified (White et al., 2007). The monitoring was performed in greenhouse, which assured that all seedlings experienced the same growth conditions.

## Materials and methods

### Provenances

The investigation was conducted on samples collected from five stands located along an altitudinal/latitudinal gradient ranging between the 48th and 53rd parallels and belonging to the coniferous boreal forest of Quebec (Canada). The sites Simoncouche (abbreviated as SIM, 48°13′ N, 71°15′ W, 338 m a.s.l.) and Bernatchez (BER, 48°51′ N, 70°20′ W, 611 m a.s.l.) were in the balsam fir [*Abies balsamea* (L.) Mill.)]—white birch (*Betula papyrifera* Marsh.) bioclimatic domain, while Mistassibi (MIS, 49°43′ N, 71°56′ W, 342 m a.s.l.) and Camp Daniel (DAN, 50°41′ N, 72°11′ W, 487 m a.s.l.) were in the black spruce-moss bioclimatic domain. Mirage (MIR, 53°47′ N, 72°52′ W, 384 m a.s.l.) was in the black spruce-lichen domain, at the boundary of the taiga subzone, where stands show lower density and growth. The five populations were even-aged, mature, closed and pure black spruce stands with 120–140 year-old trees.

The climate of the area is typically boreal, with very cold winters having absolute minimum temperatures lower than −45°C and short and cool summers. May-September mean temperatures range between 11.1 and 14.6°C, with a total precipitation of 411–675 mm. Temperatures change according to latitude and altitude, with the stands located at the higher altitudes being the coldest in winter and the least warm in summer (see Table S1 for the temperatures of each stand). No clear trend in May-September precipitation was observed among studied sites. More information about climatic conditions of the sample stands is reported in Boulouf Lugo et al. (2012) and Rossi et al. (2014).

### Cone sampling and seed extraction

Five to 10 dominant trees were randomly selected in each stand and 2–25 cones were sampled from each tree according to availability and the accessibility of the canopy, for a total of 75–145 cones per stand. Sampling was conducted in June 2012, after complete snowmelt had occurred. During seed extraction, the cones of each tree were carefully kept separate. At the end of June, the cones were placed in an oven at 80°C for 4 h to force their opening, and shaken in beakers by a Vortex mixer for 1 min with the rotating speed of the paddle set at 2500 rpm (Sirois, 2000; Simard and Payette, 2005). The seeds were manually sorted with tweezers, and any remaining seeds were removed from the cone scales by shaking batches of 2–3 cones in a mixer mill at 15 oscillations per second for 1 min. Seed extraction was performed under a stereoscope so that specimens with damaged seed coat could be removed.

### Germination

A part of the collected seeds was used for testing seed viability. The seeds were spread over filter papers in germination trays and maintained in a growth chamber at 30°C day/20°C night, with 8 h photoperiod (Rossi et al., 2012c). The germination test ran for 28 days with daily counts of germinated seeds.

### Timings of seedling emergence

In July 16th and 17th, seeds were sown in plastic containers filled with peat moss, perlite and vermiculite medium. Each container was 35 × 22 cm, with 25 cavities of 200 cm^3^ volume and 12 cm depth. The experimental design consisted of 3 random plots of 5 adjacent cavities per tree, each one containing 3 seeds. Thus, each tree was member of a half-sib family (i.e., seeds having a common and known mother tree, but unknown father tree) of 45 seeds sown in a total of 15 cavities. Containers were kept in a greenhouse at 25°C day/18°C night with 18 h photoperiod and irrigated every 2 days to field capacity. The cool summer temperatures of the region allowed to easily maintain the thermal conditions during the experiment. The enlightened portion of the greenhouse was used because black spruce is adapted to develop under post-fire conditions in open areas. Monitoring was performed daily for 25 days, recording the date of emergence of the first seedling in each cavity. The beginning of the experiment was represented by July 17th, when all containers were irrigated for the first time.

### Bud break assessment

When seedling emergence was complete and the monitoring finished, cavities with more than one seedling were thinned by gently pulling the surplus seedlings out. The containers were kept in the greenhouse until October for seedlings to complete the first year of growth, and then maintained during winter dormancy in an open field close to the greenhouse. In March 14th, the seedlings were released from snow and transferred into the greenhouse at 25°C day/18°C night with 18 h photoperiod and irrigated every 2 days to field capacity. Seedlings were examined daily for assessing the dates of apical bud break, which were reported as days from the beginning of monitoring, corresponding to the day when the seedlings were transferred into the greenhouse. Five bud break phases were defined: (1) open-elongated bud, with lengthening scales; (2) swollen bud, with smooth and pale-colored scales but no visible needle; (3) translucent bud, with needles visible through the scales; (4) split bud, with open scales but needles still clustered; and (5) exposed shoot, with needles completely emerged from the surrounding scales and spreading outwards (Dhont et al., 2010).

### Statistical analyses

The proportions of germinated seeds were compared using a Generalized Linear Mixed Model (GLMM, SAS 9.2, SAS Institute Inc., Cary, NC). GLMM was modeled with logit-link function, where the response variable was implemented as the ratio between number of events (number of germinated seeds) and number of trials (number of tested seeds). The effect of each factor to the variance of the dependent variable (bud break phases) was estimated using the Restricted Maximum Likelihood (REML) method and Generalized Linear Model (GLM). REML was conducted by considering the plot effect and by nesting the half-sib family effect within the provenance effect. Altitude and latitude of each stand were used as independent variables in linear regressions for quantifying their effects on seedling emergence and the five bud break phases.

### Exploring and testing causal models

The causal structure of the process of bud break was investigated by means of path models (Shipley, 2000). The relationships between the bud break phases were initially assessed by Pearson correlation coefficients. The resulting correlation matrix was used to search for path models that were consistent with the observations at a specified probability level. The analysis explored potential causal relationships employing the SGS algorithm as implemented in the EPA2 program (Shipley, 1997). Firstly, the algorithm explores all possible dependency links between pairs of variables when all possible subsets of other variables in the model are mathematically fixed. Secondly, the algorithm attempts to orientate the undirected dependency links between each pair of related variables. In other words, either link or independency between each variable and every other variable was analyzed, and the resulting links between variables were converted into causal relationships. The complete analytical description of the algorithm was reported in detail by Shipley (2000).

Based on the exploratory analysis, which identified all possible causality links between the dates of bud break and cell production, the causal models produced by the SGS algorithm were tested according to d-sep tests (Shipley, 2000). All constraints in the form of independence statements were identified. The number of constraints was associated to the amount and arrangement of the causal links of each model, where arrows (→) describe the causal links between variables. Let α, β, γ be three variables with α → β and no causal link between β and γ, the notation β_∥_γ|α means that β is independent of γ upon conditioning on the complete set of parents of β and γ, here represented by α. Each constraint was tested by a generalized mixed model [MIXED procedure in SAS 9.2 (SAS Institute Inc., Cary, NC)] according to Shipley (2009). Each mixed model measured the connection between the independent and dependent variables (e.g., β and γ, respectively), controlling for the effect of a third (e.g., α), by obtaining the probability that the partial regression slope of the dependent variable was zero. The exact probability level of the *j*-independence relation being *p_j_*, Shipley (2009) illustrates the procedure to verify each model as a whole according to Fisher's *C* statistic.

$$C=-2\sum_{j=1}^{k}\ln(p_{j})$$

where *k* is the number of independence relations listed for the causal model. The statistical significance of Fisher's *C* is verified by the χ^2^-probability function with 2*k* degrees of freedom (df).

## Results

### Germination

The germination percentage ranged between 9 and 57%, with the provenances BER and DAN showing the higher seed viability (Figure S1). The lowest germination rates were observed for MIS and MIR, which also exhibited smaller and lighter cones. On average, the latter provenances had cones of 1.14 g, while the cones of SIM, BER and DAN weighed 1.46 g (Figure S1). The statistical analysis confirmed the difference in germination rate between provenances (GLMM, Wald χ^2^ = 30.11, *P* < 0.0001). However, when the weight of the cones was included as a co-factor in the analysis, the results changed markedly: the effect of the provenance became non-significant (GLMM, Wald χ^2^ = 7.50, *P* = 0.11), while cone weight had significant effects on germination (GLMM, Wald χ^2^ = 22.09, *P* < 0.0001). The interaction between factors was also significant (GLMM, Wald χ^2^ = 14.76, *P* < 0.01), indicating that the effect of the provenance depended on the cone weight. Although statistics were conducted on the proportion, the results for MIR should be considered with caution because of the smaller amount of seeds available for the analysis from this provenance.

### Timings of seedling emergence

The period of seedling emergence began 8 days after sowing and lasted 6 days (Figure S2). The distribution was clearly bell-shaped and normal, with the exception of MIS, which showed a higher variability. On average, the seedlings appeared 10–11 days after sowing. The effects of altitude and latitude resulted in *F*-values of 2.96 and 0.41, respectively, with *P* > 0.05, which indicated no significant effect on the timing of seedling emergence.

### Bud break

**Figure 1:**
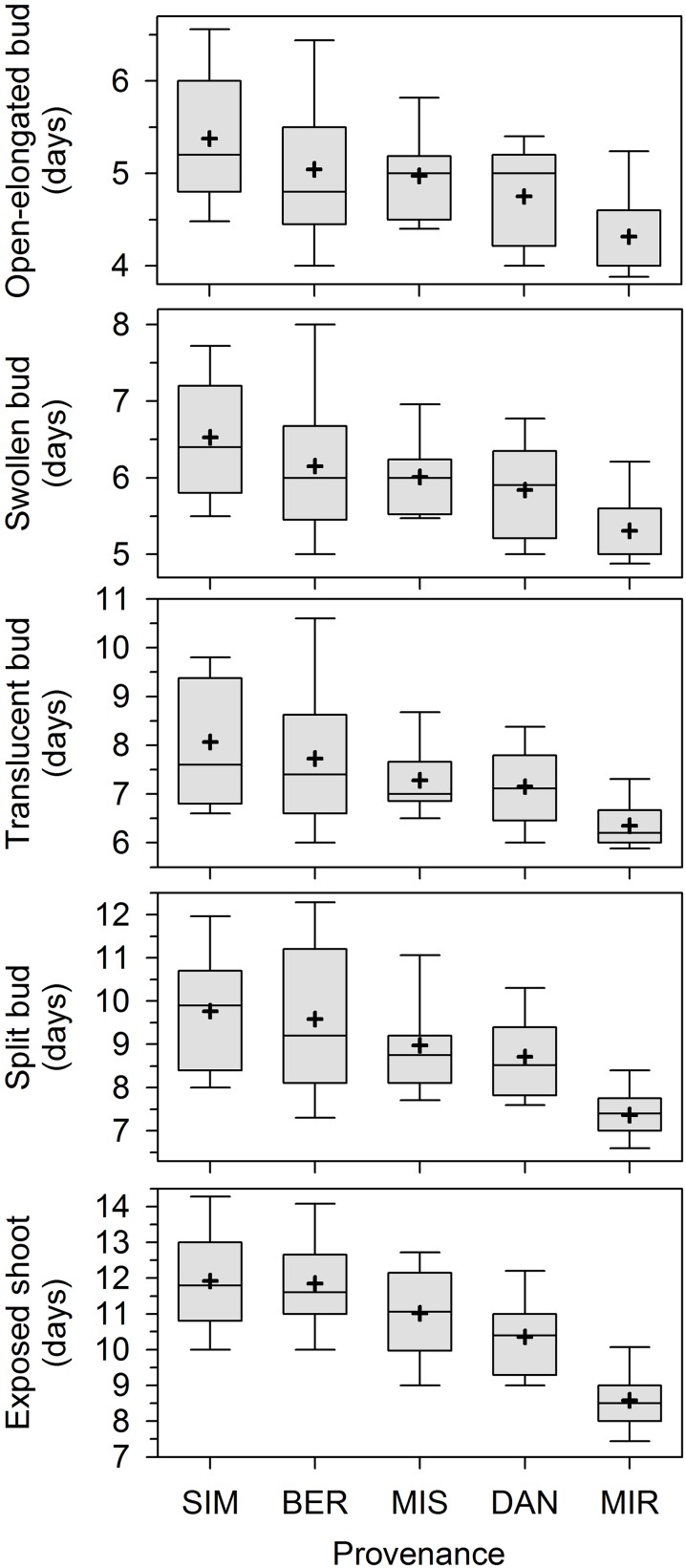
**Phases of apical bud break in seedlings derived from seeds collected in five stands of the closed black spruce forest of Quebec, Canada**. Bud break is reported as days after the beginning of the monitoring, which corresponded to the transfer of plants into the greenhouse. Provenances are listed according to increasing latitude of the stands of origin. Boxes represent upper and lower quartiles, whiskers achieve the 10th and 90th percentiles and the mean and median are drawn as cross and horizontal solid lines, respectively.

Overall, apical bud break in our experiment lasted 15 days. The first open-elongated bud was observed 3 days after the beginning of monitoring, and this first phase was completed within 10 days (Figure 1). The successive phases occurred later and partially overlapped among individuals. The provenances showed different phenologies, with earlier timings of bud break being clearly observed in seedlings of MIR. Bud break occurred later in SIM and BER.

The contribution of the various factors to the total variance was quite similar for the five phenological phases (Table 1). The effect of all factors was significant at *P* < 0.05 or lower. The factor provenance explained between 10.2 and 32.3% of the variance, the higher percentage being observed for the last phase (exposed shoot). The percentage of the variance accounted for by half-sib families within provenances was smaller but statistically significant, ranging between 3.9 and 9.5%. The differences between plots in the greenhouse explained between 3.0 and 7.8% of the variance, indicating low to moderate environmental variation within the greenhouse. The residual variance was the highest (61.3–78.1%), particularly for the first phenological phases.

**Table 1:** **Components of the variance of the five phases of bud break and respective significance**.

**Phenological phase of bud break**	**Provenance**	**Half-sib family within provenances**	**Plot**	**Residual**
Open-elongated bud	0.12 (10.2)^***^	0.12 (9.5)^***^	0.04 (3.0)^**^	0.95 (77.3)
Swollen bud	0.17 (10.3)^***^	0.13 (8.0)^**^	0.06 (3.5)^**^	1.26 (78.1)
Translucent bud	0.34 (10.7)^***^	0.25 (7.9)^***^	0.24 (7.8)^***^	2.31 (73.6)
Split bud	0.81 (18.5)^***^	0.35 (7.9)^***^	0.23 (5.3)^***^	2.98 (68.3)
Exposed shoot	1.79 (32.3)^***^	0.22 (3.9)^*^	0.14 (2.5)^**^	3.40 (61.3)

*Percentage contributions of each effect are reported in parentheses*.

* *P < 0.05*,

** *P < 0.01*,

*** *P < 0.001*.

**Figure 2:**
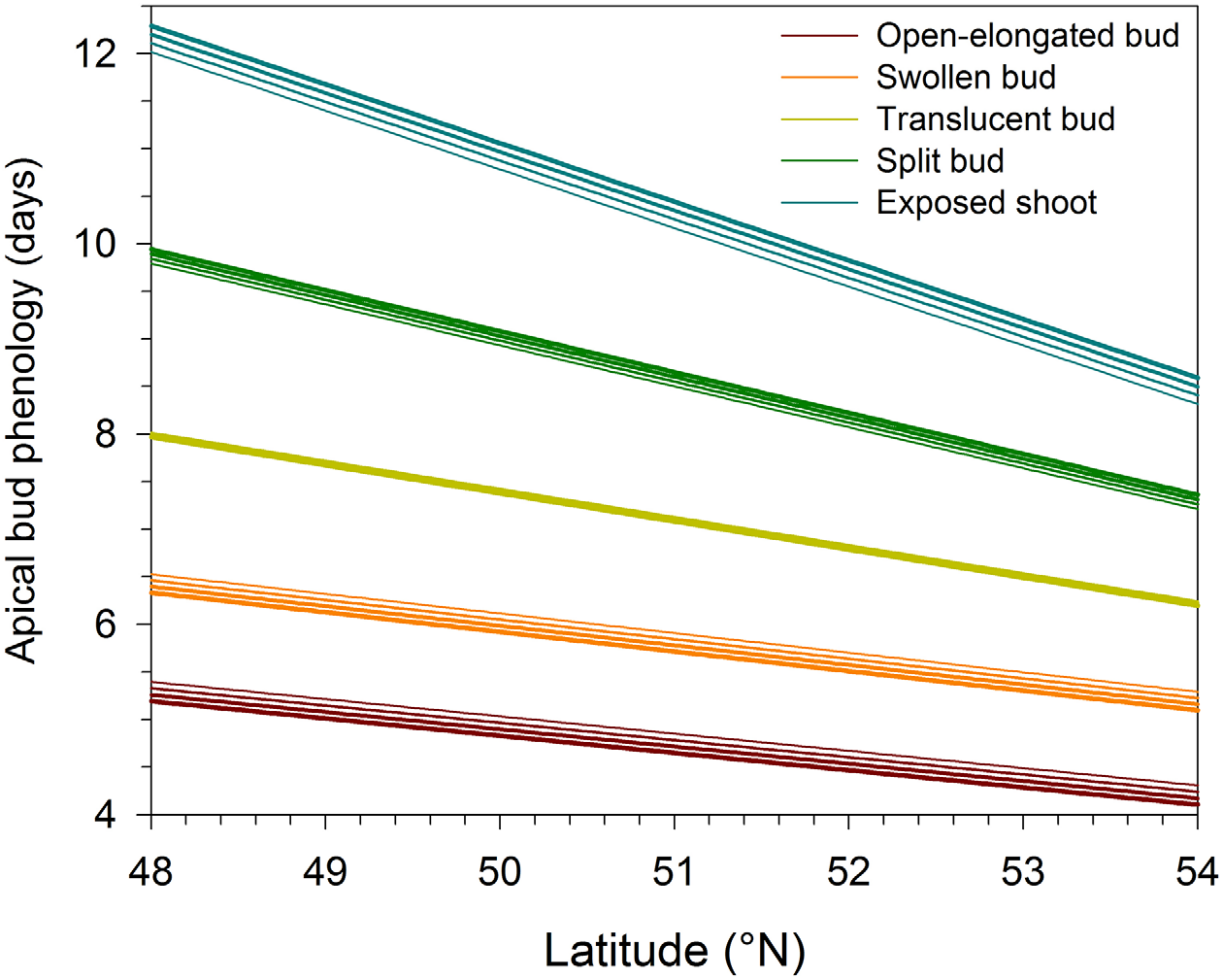
**Variation in the phases of apical bud break in black spruce seedlings estimated by linear regressions in function of latitude and altitude**. Bud break is reported as days after the beginning of the monitoring, which corresponded to the transfer of plants into the greenhouse. Line thickness represents altitudes varying between 300 (thinner line) and 600 (thicker line) m a.s.l.

The regressions implicating altitude and latitude of provenances were highly significant (*P* < 0.0001), resulting in *R*^2^ ranging between 0.21 and 0.51 (Table 2). The Studentized residuals of the regressions showed no trend and were uniformly distributed around zero, suggesting that the analysis could be considered reliable (Figure S3). The low variance accounted for was due to the variability observed between the seedlings within each provenance. However, no significant difference was observed when comparing the phenology between half-sib families, indicating that seedlings belonging to different trees of the same provenance exhibited similar timings of bud break (data not shown). Latitude significantly affected the five phases of apical bud break, as shown by the low probabilities of *t*-values (*P* < 0.0001). For all regression models, the effect of altitude on bud break was not significant (*P* = 0.05). The timings occurred earlier at higher latitudes, with the latest phases of bud break gradually exhibiting the greatest coefficients in absolute value (Table 2). Accordingly, one additional degree of latitude corresponded to an anticipation of the timings of open-elongated bud of only 0.18 days, but to an anticipation of the timings of exposed shoot of 0.61 days. Consequently, seedlings of northern latitudes exhibited an earlier and quicker development of apical buds as predicted by the regressions shown in Figure 2 and calculated using the coefficients estimated in Table 2.

**Table 2:** **Results of the regression models for the five phases of apical bud break measured in seedlings derived from seeds collected in five stands of the closed black spruce forest of Quebec, Canada**.

**Phenological phase**	**Model**		**Coefficients**			***t*-value**		
	***F*-value**	***R^2^***	**Intercept**	**Altitude**	**Latitude**	**Intercept**	**Altitude**	**Latitude**
Open-elongated bud	15.56*	0.22	14.30	−6.77 × 10^−4^	−0.18	8.37*	−1.11	−5.58*
Swollen bud	17.08*	0.24	16.60	−6.43 × 10^−4^	−0.20	8.97*	−0.97	−5.84*
Translucent bud	14.41*	0.21	22.24	−1.63 × 10^−4^	−0.29	7.60*	−0.16	−5.30*
Split bud	24.64*	0.31	30.29	4.97 × 10^−4^	−0.43	9.11*	0.42	−6.80*
Exposed shoot	56.27*	0.51	41.34	9.21 × 10^−4^	−0.61	13.05*	0.81	−10.23*

*Asterisks indicate values significant at P < 0.0001. Other values not significant (P = 0.05)*.

### Exploring and testing causal models

**Figure 3:**
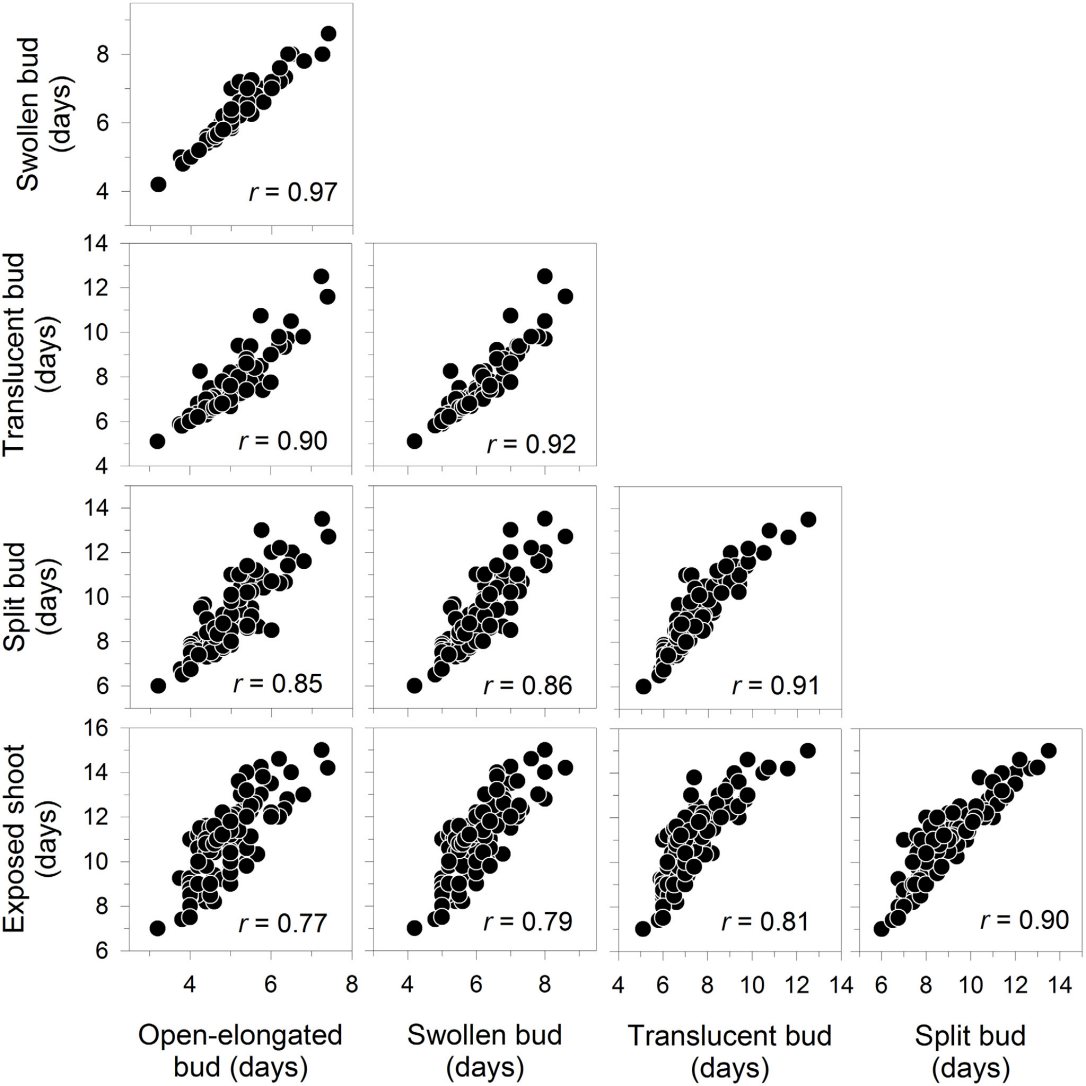
**Correlation matrix between the dates of the phases of apical bud break in black spruce seedlings**. All correlation coefficients are significant at *P* < 0.001. Bud break is reported as days after the beginning of the monitoring, which corresponded to the transfer of plants into the greenhouse.

All correlations between the dates of bud break were positive and significant at *P* < 0.0001, with values between 0.77 and 0.97 (Figure 3). Accordingly, the late occurrence of one phenological phase corresponded to a delay in the other phases. The relationships followed a linear pattern within the analyzed range.

**Figure 4:**
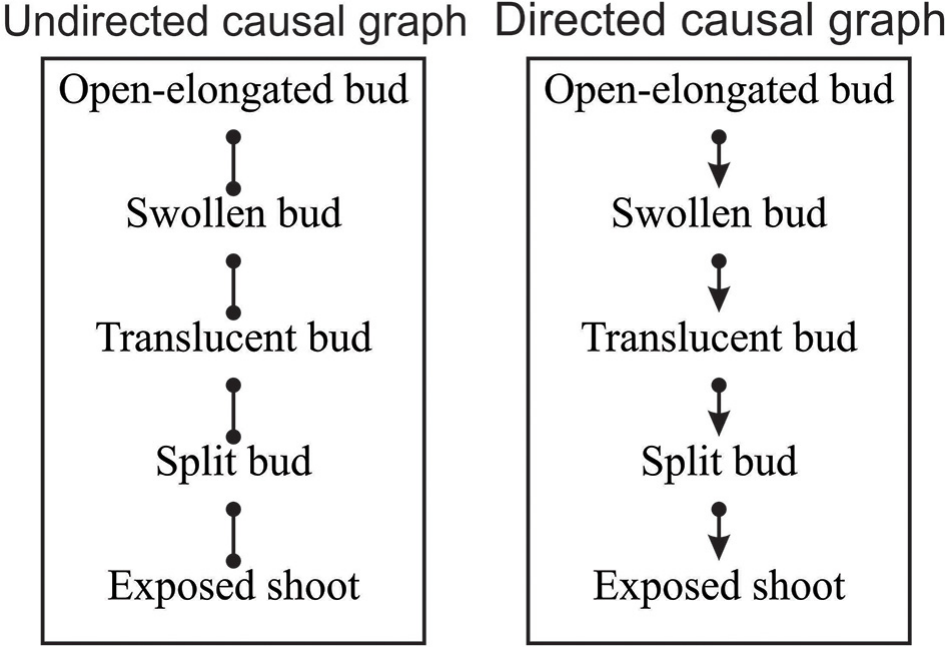
**Undirected and directed causal graphs of the dates of bud break**. Dots represent a symmetrical association between variables while arrows indicate the causal links between variables, where α → β indicates that α affects β.

According to the combinatorial computation, 1,048,576 potential causal models could be defined using the five variables (Shipley, 2000). Among these, EPA2 program found eight possible dependency links between pairs of variables that were not rejected by the SGS algorithm and could be valid. The resulting potential causal model consisted of an undirected dependency graph where the lines connected the two probabilistically dependent variables conditional on every subset of other variables in the graph (Figure 4 on the left). In its present form, the relationships identified by the lines express a symmetrical association between pairs of variables.

The symmetrical association between variables of the undirected causal graph were then converted into asymmetrical causal relationships as illustrated by the directed causal graph in Figure 4 (on the right), where arrows describe the causal links between variables (e.g., α → β indicates that α affects β) and specify how causal effects could propagate through the variables. For the identification of the causal relationships, the temporal rule in which effects might propagate only from one phenological phase to the next was assumed. According to the directed causal graph, each phase was linked to the previous and successive one, while no causality was observed between non-adjacent phases (Figure 4). The causal relationships entailed five independence constraints, which corresponded to mixed models generating *F*-values of between 0.09 and 1.37 and *P* = 0.05 (Table 3). Fisher's *C* statistic calculated on these constraints was 9.94. The χ^2^-probability function with 10 df corresponded to a *P* = 0.44. The d-sep test was not significant and unable to reject the proposed model. According to these findings, the directed causal graph described in Figure 4 should be considered statistically valid.

**Table 3:** **Independence constraints and relative statistical verification in terms of significance of the partial regression slope assessed using generalized mixed models (*F*-value), degrees of freedom (df) and Fisher's *C* statistic for the directed causal graph involving the dates of bud break [open-elongated bud (P1), swollen bud (P2), translucent bud (P3), split bud (P4) and exposed shoot (P5)]**.

**Constraints of the directed causal model**	**Statistical verification of the constraints**
P1_∥_P3|P2	*F*_P1,P3|P2_ = 1.37
P1_∥_P4|P3	*F*_P1,P4|P3_ = 1.22
P1_∥_P5|P4	*F*_P1,P5|P4_ = 0.96
P2_∥_P4|{P1,P3}	*F*_P2,P4|P1,P3_ = 0.09
P2_∥_P5|{P1,P4}	*F*_P2,P5|P1,P4_ = 0.67
	*df* = 10
	Fisher's *C* = 9.94

*The notation β_∥_γ|α means that β is independent of γ upon conditioning on the complete set of parents of β and γ, here represented by α. All results are not significant (P = 0.05)*.

## Discussion

### Timings and dynamics of bud break

In this study, the phenological phases of bud break were monitored daily on black spruce seedlings growing from seeds collected along an altitudinal/latitudinal gradient in the closed black spruce forest of Quebec, Canada. Although no difference was observed in the percentage of seed germination and timings of seedling emergence, the five provenances showed different bud break timing. Seedlings from higher latitudes had earlier bud break, while in all models, the effect of altitude was not significant. These results were in part expected because provenance variation in bud break has been observed in the sympatric white spruce (Li et al., 1997), and because provenance variation in another important phenological trait, bud set, had been previously reported in black spruce (Beaulieu et al., 2004). The results obtained conform to the general view that northern provenances generally require less heat accumulation for bud break than southern provenances (Blum, 1988). Phenology is a balancing act between growth maximization and frost damage avoidance (Chuine, 2010). In late spring, when day length is longer, nights are short and frost events are unlikely to occur. The earlier breakage of dormancy observed in the northern provenances thus appears to reflect an adaptation to cold environments, allowing growth to be resumed as soon as possible and thus lengthening the growing season. The lack of significant results for altitude could be related to the small range provided by the sites (273 m of difference in altitude calculated between the highest and lowest site).

Latitude was observed to have different effects on the successive phases of bud break, with the later phases being proportionally more anticipated than the earlier ones at higher latitudes. As a result, the development of the apical bud in seedlings from northern latitudes was quicker than that in seedlings from southern latitudes. At high latitudes, the trees are adapted to produce new tissues in colder conditions, and the physiological processes involved in bud development are more rapid than in southern provenances when compared at the same temperature (Körner, 2003). Genotypes of cold climate combine high metabolic activity with a strict developmental regime, according to a genetically-engraved evolutionary strategy that maximizes security (Körner, 2012). Earlier and quicker phenological events in spring would provide greater advantages for plants than later autumnal events because spring is more closely linked with favorable conditions for photosynthesis and growth, especially at higher latitudes, where photoperiod decreases abruptly at the end of summer (Rossi et al., 2006; Chuine, 2010).

### Structure of the leafing process

A comprehensive exploration of the relationships between bud break phases was conducted in this study, which identified the most likely scenario among all potential models. Like most growth activities, bud break is a continuous process that can be partitioned in discrete elements, composed by arbitrary but objectively recognizable phases (Dhont et al., 2010). According to the models tested, which represented the most parsimonious hypothetical and confirmatory model, leafing in black spruce appeared as a linear chain of events producing effects on the contiguous phases where the timings of occurrence of each phase influence those of the successive one. The absence of relationships connecting non-contiguous phases indicated that the simple temporal succession of the phenological events was sufficient to describe the structure of the leafing process. These findings diverge from previous observations on secondary meristems that demonstrated a higher complexity of xylem phenology, which appeared like a network rather than a linear chain (Rossi et al., 2012a). However, such differences could be related to the number and nature of the variables considered by the model. Rossi et al. (2012a) used more variables covering all phases of the growing season, including those at the beginning and end of growth. In the present analysis, only the phenological phases occurring in spring were considered. These results did not arise from a direct manipulation under controlled experiment and all causal inferences were tested using a well-acknowledged statistical procedure (Shipley, 2009). However, the resulting model, based on real observations, suggested that the bud break process could have a precise and recognizable framework that could be verified with specific experiments. Such a framework could also be useful in interpreting and clarifying the relative importance of endogenous and environmental factors affecting the bud break process.

### Adaptation at the regional scale

Wide geographical distributions require the ability to grow in a large range of environmental conditions. This ability is revealed by plasticity but also by the genetic variation in phenology and growth among provenances or populations of a same species (Beaulieu and Bousquet, 2010). In the eastern part of the black spruce natural distribution where this work was carried out, this variation in growth has been considered as an ecotypic differentiation (Khalil, 1975). The moderate but significant differences in the timings of bud break that were observed among provenances of black spruce suggest that genetic adaptation to local conditions exists even at regional scale.

In conifer species of the boreal forest, most genotypic variation is usually detected within rather than among populations, which has been ascribed to the short period allowed for evolutionary adaptation following the post-glacial colonization and the intense gene flow occurring among populations (Beaulieu and Bousquet, 2010; Prunier et al., 2011). In the present study, while the family-within-population effect was moderate, the residual variance still retained most of the total variance, ranging from 61.3 to 78.1%. Such proportions indicated a high variability in the timings of all bud break phases between the seedlings belonging to the same half-sib family. A similar pattern was seen in white spruce (Li et al., 1993). While this variance might be caused in part by maternal effects related to seed size and nutritional reserves (carry-over effects) as well as micro-environmental variation in the experimental design, it also likely reflects genetic variation among half-sibs of the same family (Perry and Bousquet, 2001). Such a reservoir of genetic variation within populations could be highly valuable in connection with the adaptive capacity of local populations to genetically evolve in the next generation in response to changes in environmental conditions that might exceed the limits of physiological plasticity.

### Bud break and climate change

In the last century, an increasing trend has been observed in the global temperature, with the higher latitudes experiencing the greatest rates of warming than most other regions of the world (IPCC, 2013). The regional models predict an exacerbated rate of warming under the latitudes of the present sampling (Houle et al., 2012). The variation observed in the dynamics of bud break and the delay accumulated during the successive phases from swelling buds to exposed shoots and needles could play an important role in the responses of species with wide geographical distributions to the current warming trend. According to Andalo et al. (2005), populations of high latitudes, where growing seasons are shorter and growth quicker, could be expected to be more sensitive or responsive than the slower growing southern populations. Evidence supporting this hypothesis is already available for primary and secondary meristems (Sparks and Menzel, 2002; Boulouf Lugo et al., 2012). The latitudinal pattern of warming has been associated to the different degrees of anticipation of leafing dates between Northern and Central Europe (Sparks and Menzel, 2002). The current resumption of cambial activity and xylem formation in black spruce has been estimated to occur 2.5–4.0 days early than in 1950s, with the greatest changes in the northern stands (Boulouf Lugo et al., 2012).

In this study, black spruce provenances were compared in a greenhouse that provided the artificial and uniform growing conditions for allowing a better expression of growth potential and differences among populations. However, when bud break occurs in the field, snow has just disappeared or is still melting, and black spruce experiences a slower warming, lower air temperatures and freezing soil conditions (Rossi et al., 2009, 2011). Photoperiod is also known to interact with temperature in affecting bud break (Partanen et al., 2001; Rossi, 2014), thus the constant day length set in this experiment does not represent a natural condition. Moreover, ontogenic changes in phenology are often observed during the tree lifespan, with different gene polymorphisms implicated in genetic variation at different ages (Prunier et al., 2013) and therefore, different responses may be expected in mature individuals (Partanen et al., 2001). Nevertheless, the greenhouse allowed a reliable control and uniformity of the environment during the experiment so that genetic effects could be disentangled in part from physiological and environmental variation. Consequently, any ecological interpretation and implication should be taken with caution and await confirmation from more complete investigations or long-term observations in the field (Körner, 2012).

## Conclusions

Leafing of black spruce is composed by a distinctive chain of events producing effects on the contiguous phases, where the timings of occurrence of each phase influence those of the successive one. Bud break of seedlings well reflected the latitude of their origin, confirming the genetic signature of evolutionary adaptation of black spruce populations to their local temperature conditions. The latitudinal differences in bud break were related to both timings and dynamics. Accordingly, when compared at the same temperature conditions, the provenances of high latitudes had an earlier resumption of bud break associated to quicker accomplishment of all phenological phases, which resulted in an earlier leafing. This study demonstrated that the evolutionary adaptation of populations of high latitudes points toward a survival strategy based on physiological processes that are quick and triggered by critical heat accumulation. As observed for other growth traits in trees of cold environments, the genetic variation in bud break of black spruce along its latitudinal distribution integrates high metabolic activity with maximization of security as result of a combination of gene flow and strength of local selection.

The findings of this work provided evidence of the genetic adaptation in phenology of black spruce populations to their local environmental conditions and how the pattern of variation in bud break occurs among and within populations along the entire latitudinal range of the closed black spruce forest in Quebec, Canada. Despite the optimal control of artificial growing conditions used and although the high resolution of the monitoring produced reliable results, these findings should be tested for confirmation in older individuals because of the demonstrated ontogenic differences in phenology between young and mature trees. Although variation among provenances was sizeable, much variation was also detected within populations. This local genetic diversity represents a potent reservoir of adaptation for the next generations in addition to the phenotypic plasticity, allowing the future natural regeneration of black spruce to possibly adapt to different environmental conditions.

### Conflict of interest statement

The authors declare that the research was conducted in the absence of any commercial or financial relationships that could be construed as a potential conflict of interest.
