# Supplementary material for: The bud break process and its variation among local populations of boreal black spruce
Source: Front Plant Sci. 2014 Oct 28;5:574. doi: 10.3389/fpls.2014.00574 (PMC4211384; doi:10.3389/fpls.2014.00574)
Supplement: Supplementary file 1 [file DataSheet1.DOCX]

# Disentangling genetic from environmental components of the Bud break process in black spruce

## Sergio Rossi and Jean Bousquet

|  |  | Annual temperature (°C) | | |
| --- | --- | --- | --- | --- |
| ID | Site | Mean | Absolute  maximum | Absolute  minimum |
| SIM | Simoncouche | 4.1 | 31.0 | -29.8 |
| BER | Bernatchez | 1.8 | 29.6 | -36.5 |
| MIS | Mistassibi | 2.0 | 32.1 | -36.9 |
| DAN | Camp Daniel | 0.0 | 30.6 | -42.7 |
| MIR | Mirage | -1.6 | 30.0 | -47.1 |

**Table S1** Mean and absolute temperatures recorded in the five black spruce stands where seeds were collected.

|  |
| --- |

**Figure S1:** Average cone weight and germination percentage of seeds from the five provenances of the boreal forest of Quebec, Canada. Error bars correspond to the standard deviation.

|  |
| --- |

**Figure S2:** Timings of emergence of seedlings derived from seeds collected in five stands of the closed black spruce forest of Quebec, Canada. The dates are reported as days after sowing. Boxes represent upper and lower quartiles, whiskers achieve the 10th and 90th percentiles and the mean and median are drawn as cross and horizontal solid lines, respectively.

|  |
| --- |

**Figure S3:** Studentized residuals vs predicted values resulting from the regression models performed on the dates of apical bud break according to latitude and altitude of the sites.
